# Ada2 acts upstream of Pdr802 in regulating macrophage-enhanced virulence of *Cryptococcus neoformans*

**DOI:** 10.1128/spectrum.01398-25

**Published:** 2025-08-11

**Authors:** Tyler J. Cernohous, Laura C. Ristow, Mark A. Stamnes, J. Muse Davis

**Affiliations:** 1 Department of Biology, University of North Dakota 169224, Grand Forks, North Dakota, USA; 2 Stead Family Department of Pediatrics, Carver College of Medicine, University of Iowa 12243 , Iowa City, Iowa, USA; 3 Department of Molecular Physiology and Biophysics, Carver College of Medicine, University of Iowa 12243 , Iowa City, Iowa, USA; Virginia-Maryland College of Veterinary Medicine, Blacksburg, Virginia, USA

**Keywords:** Cryptococcus, macrophage, pathogenesis, autophagycytosis

## Abstract

*Cryptococcus* is the leading cause of fungal meningitis in humans and a major source of mortality in people living with HIV. Infection starts in the lungs and results in meningoencephalitis after dissemination via the bloodstream. Alveolar macrophages are the first immune cells to encounter the infection and are usually capable of clearing it. In immunocompromised hosts, *Cryptococcus* survives and grows inside macrophages, establishing pulmonary and ultimately central nervous system infection. The initial interaction with macrophages is a key event in pathogenesis, and we have demonstrated that macrophage exposure induces an enhanced virulence phenotype in *Cryptococcus*. This process can be replicated *in vitro*: yeast cells exposed to macrophages in culture demonstrate enhanced virulence in both zebrafish and mouse models. To understand this induction process, we investigated its effect on the cryptococcal transcriptome to determine what cryptococcal factors are required. Ada2 is a subunit of the transcription modulating Spt-Ada-Gcn5-acetyltransferase complex and is required for cryptococcal virulence. We find that a cryptococcal strain lacking Ada2 is not capable of enhanced virulence after macrophage exposure. We further show that Pdr802, a transcription factor with a significant but poorly understood role in cryptococcal pathogenesis, acts downstream of Ada2 during macrophage exposure, and loss of Pdr802 function is sufficient to block macrophage-enhanced virulence. These findings shed light on the earliest stages of cryptococcal pathogenesis and offer new clues to the functions of Pdr802.

**IMPORTANCE:**

*Cryptococcus* is a fungal pathogen affecting mostly immunocompromised people, especially those with HIV. Infection starts in the lungs but ultimately disseminates to cause meningitis, leading to over 50% mortality in patients with AIDS. Cryptococcal meningitis is difficult to treat, and understanding the early stages of infection will be the first step toward preventing it. Lung macrophages are the first immune cells to encounter *Cryptococcus*, and while they clear the infection in some cases, in immunocompromised patients, they instead serve as a niche for cryptococcal growth. How can macrophage interaction promote infection? We have shown that in a susceptible host, cryptococcal virulence is increased after initial contact with macrophages. This effect can be replicated *in vitro*, as cryptococcal cells pre-exposed to cultured macrophages are more virulent in animal models. We have identified two cryptococcal genes that regulate this change in virulence, setting the stage for a better understanding of cryptococcal pathogenesis.

## OBSERVATION

Cryptococcal infections account for a substantial amount of morbidity and mortality among immunocompromised populations. *Cryptococcus* (Cn) tops the list of fungal priority pathogens published by the World Health Organization in 2022 (1). This fungus is ubiquitous in the environment, and pathogenesis begins with inhalation into the lungs. Cn not cleared by macrophages moves out of the airways and into the parenchyma, where it persists in both intra- and extracellular niches. Uncontrolled growth in the lung leads to bloodborne dissemination to the central nervous system. The resulting meningoencephalitis can be difficult to detect before it becomes very difficult to treat. A longstanding dilemma in Cn pathogenesis concerns the role of macrophages: there is evidence to support both host-protective and host-detrimental roles for the types of macrophage found in the lung (2–4).

Using the zebrafish model, we previously showed that Cn is initially and completely phagocytosed and that pathogenesis only proceeds after hours to days of intra-phagocyte residence (5). We subsequently demonstrated that pre-exposure to cultured macrophages leads to enhanced Cn virulence *in vivo* (6). We have termed these Cn cells macrophage exposed cells (MECs), and here we have sought to understand the cryptococcal factors required for their virulence.

**Fig 1:**
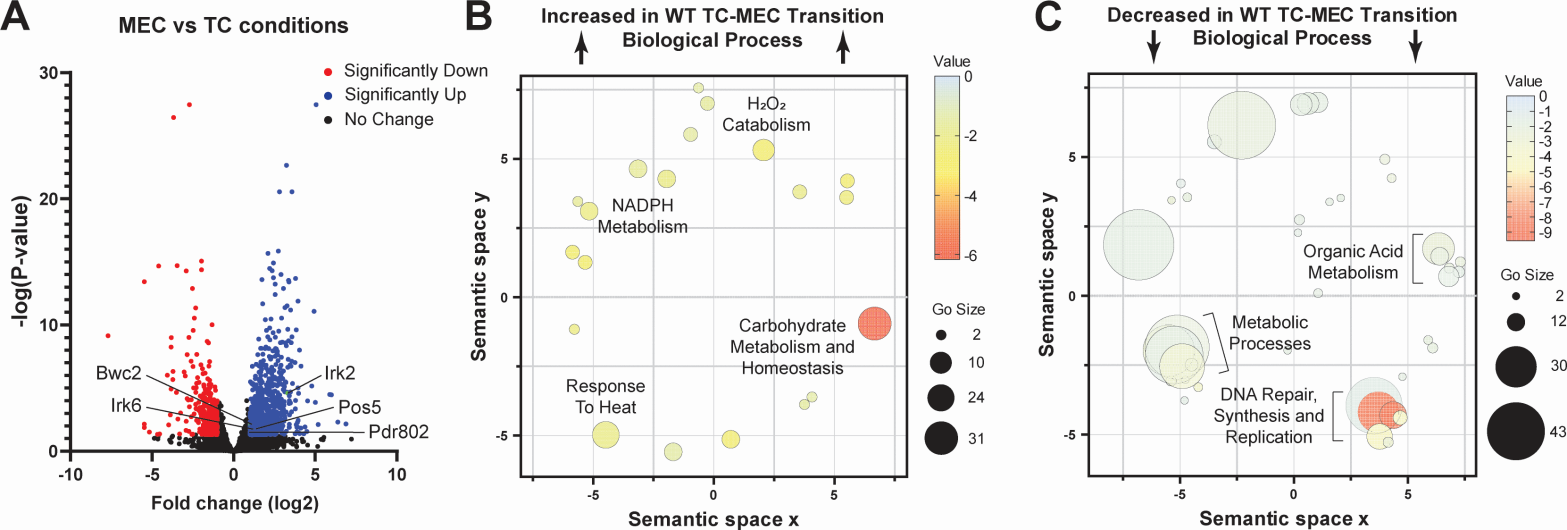
Transcriptomic analysis of the TC to MEC transition. (A) Volcano plot of genes identified by RNA-seq in H99s co-incubated for 24 h with J774A.1 cells compared to H99s incubated in matched culture conditions alone. Source data and specific *P* values (Wald test *P* values with Benjamini-Hochberg) are reported in Data S1. (B and C) Biological function GO terms for differentially expressed genes (±log2 1 and FDR < 0.05, Benjamini-Hochberg) represented in semantic similarity scatterplots for increased and decreased genes, respectively.

First, we used RNA-seq to characterize the transcriptional differences between MECs derived from H99 (heretofore referred to as WT) and H99 incubated in identical tissue culture conditions without macrophages (TC controls). A volcano plot summarizing these data is shown in Fig. 1A. Among the upregulated genes (±1 log2 ; FDR < 0.05) were five transcription factors and kinases with known roles in virulence: Irk6, Pdr802, and Pos5 (core virulence) plus Bwc2 and Irk2 (lung stage virulence) (7). We then performed GO term analysis of all regulated genes. Consistent with the known features of the phagocyte environment, genes involved in reactive oxygen species tolerance, acquisition and use of energy sources, and response to adverse environmental factors were enriched (FDR < 0.05, Benjamini-Hochberg) (Fig. 1B). Downregulated genes were involved in processes used less under stress conditions, such as DNA synthesis and organic acid metabolism (Fig. 1C). Cellular component-based GO analysis yielded complementary results (Fig. S1A and B), in particular reflecting upregulation of cell membrane-localized factors such as virulence-associated metal transporters *CFT1*, *CIG1*, and *CTR4* (8, 9).

**Fig 2:**
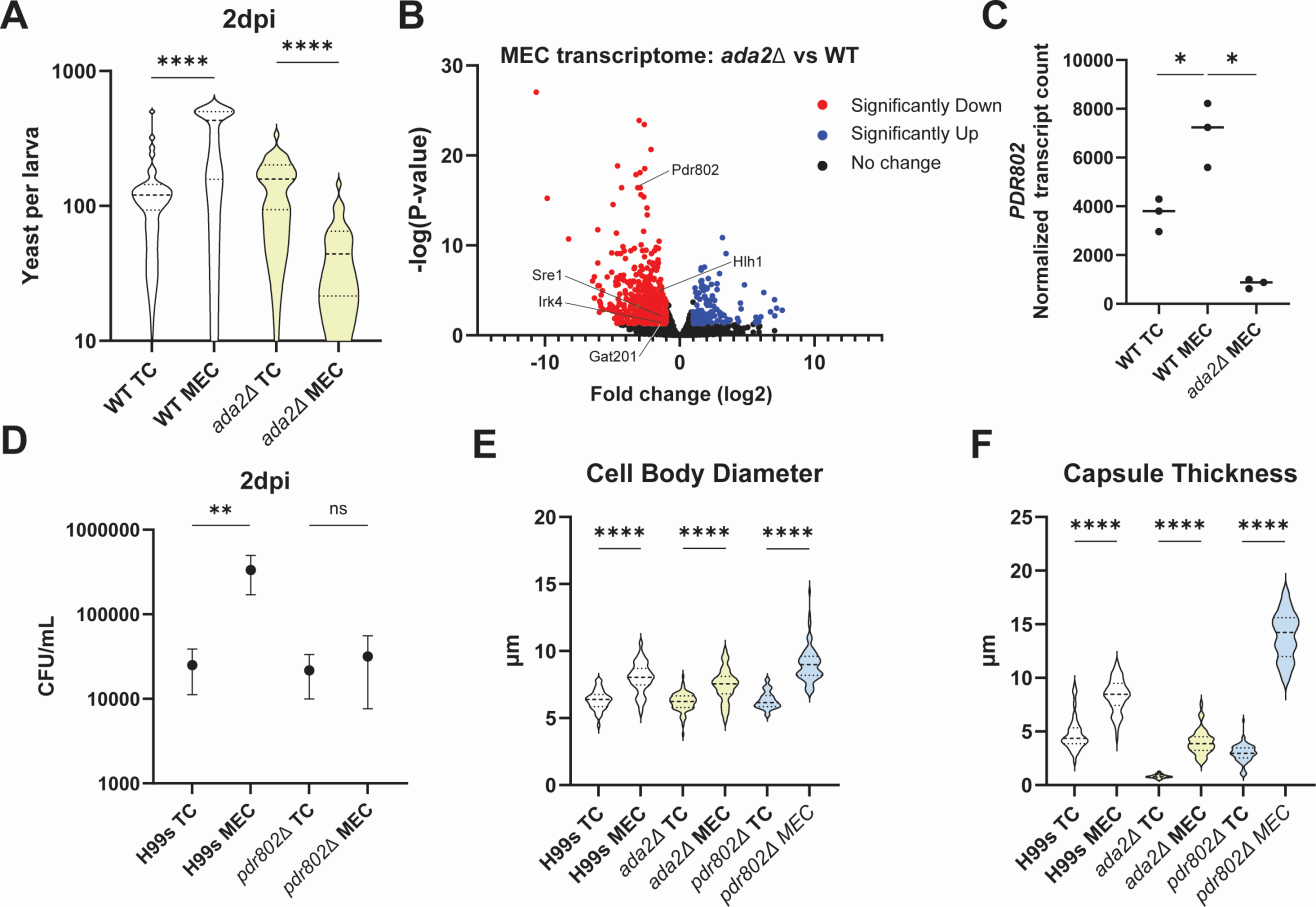
(A) Fluorescent H99s and mutant strain *ada2*∆ yeast per larva at 2 dpi as counted manually. Data from three replicates of 10 larvae each. Results of the Mann-Whitney test are shown. (B) Volcano plot of genes identified by RNA-seq of H99s co-incubated for 24 h with J774A.1 cells compared to *ada2*∆ mutant under the same conditions. Source data and specific *P* values (Wald test *P* values with Benjamini-Hochberg) are reported in Data S1. (C) Normalized transcript count from RNA-seq analysis comparing expression of *PDR802* in WT and *ada2*∆ mutant under conditions indicated. Results of Welch’s *t*-test are shown. Source data reported in Data S1. (D) Cryptococcal CFU per pool of 10 infected larvae at 2 dpi, representative replicate of WT and *pdr802*∆ mutant from conditions shown. Results of Welch’s *t*-test are shown. (E and F) Cell body diameter (E) and capsule thickness (F) for TC-conditioned and J774-transformed (MEC) strains as indicated. Measurement via widefield microscopy of India ink preparations. Data from three biological replicates of at least 20 cells per condition per replicate. Statistical comparisons represent results of two-tailed unpaired *t*-tests.

Lee et al. identified an array of regulatory genes required for Cn virulence at specific stages of pathogenesis (7). Since the initial encounter with macrophages occurs in the lung, we were surprised to find that only two lung-stage factors (Bwc2 and Irk2) were upregulated during MEC induction. We reasoned that other regulators could be playing a significant role without being transcriptionally regulated. To identify such genes, we sought a mutant strain incapable of MEC induction for further analysis. An ideal candidate gene would have an established role in virulence but broad regulatory effects. Ada2 is a component of Spt-Ada-Gcn5-acetyltransferase (SAGA), a large, multi-subunit protein complex and transcriptional co-activator with both histone acetyltransferase and de-ubiquitinase activities (10). This complex has a broad influence over the overall transcriptome. In Cn, the absence of Ada2 leads to decreased virulence in the mouse and poor growth in several stress stimuli *in vitro* (11, 12). To determine if MEC induction requires Ada2, we generated a fluorescent strain of the *ada2*∆ knockout and quantified its virulence after macrophage exposure by inoculation into zebrafish larvae and direct counting of labeled cells at 2 dpi (Fig. 2A). While TC-conditioned parental H99 and *ada2*∆ were similar in virulence, macrophage exposure reduced the virulence of the *ada2*∆ mutant. Because of the known *in vitro* fitness defects of the *ada2*∆ mutant, we tested whether the difference in virulence was due to killing during macrophage exposure. Surprisingly, the *ada2*∆ mutant survived macrophage exposure better than WT (Fig. S1C). While Ada2 is not significantly up- or downregulated in the TC to MEC transition (data not shown), this does not preclude it from being necessary for the process. Further, components of a complex as broadly active as SAGA likely exert regulatory control without changes in their own transcription.

In order to identify factors downstream of Ada2 with roles in MEC induction, we again used RNA-seq to compare MECs derived from parental H99 versus the *ada2*∆ mutant. A volcano plot of the results is shown in Fig. 2B. Downregulated virulence regulators included Pdr802 and Gat201 (core virulence), Hlh1 and Irk4 (lung stage virulence), and Sre1 (ergosterol production). Among these, Pdr802 is most promising in that it is upregulated in the WT MEC transition but not similarly expressed in MECs from the *ada2*∆ mutant (Fig. 2C). In order to determine if these candidate regulators are required for the MEC phenotype, we developed a streamlined workflow using CFU quantification of pooled infected larvae. A representative biological replicate for *pdr802*∆ is shown in Fig. 2D (two more biological replicates are shown in Fig. S1D). Pdr802 is a negative regulator of capsule production (13, 14), a key Cn virulence trait. Previously, we have shown that while macrophage exposure induces increased capsule production, this does not prevent MEC phagocytosis *in vivo* (6). Analysis of cell body and capsule size of MECs generated by the *ada2*∆ and *pdr802*∆ mutants showed that both strains increase their capsule production significantly, and the latter produces even more capsule and larger cell bodies than WT MECs (Fig. 2E and F). These data provide further evidence that while capsule production is enhanced in MECs, it is not the driving factor of their virulence.

As a regulator of the MEC phenotype, Pdr802 is of particular interest because it is highly expressed during infection (7) and required for virulence in the mouse (13–17), but its specific contribution is not clear. Three unique *pdr802∆* mutant strains have shown no deficiencies in several *in vitro* assays, including thermal tolerance, capsule, melanin and urease production, and reactive oxygen species tolerance (14, 15, 17). Paradoxically, it is a negative regulator of several functions expected to promote virulence, including capsule production, Titan cell formation, and intracellular ATP production (13, 14, 17).

The *pdr802*∆ mutant strain does show deficiencies in more complex assays. It is unable to grow in the brain after intracranial inoculation and is deficient in adherence and translocation in an *in vitro* model of the blood-brain barrier (7). Exactly how regulation by Pdr802 could contribute to these phenotypes is unclear. In the mouse model, the subpopulation of Cn most likely to disseminate features a relatively small cell body size and thin capsule (18), which may be in part due to Pdr802 inhibition of cell body size and capsule formation. More work will be required to connect this potential role with macrophage exposure. Another area for future investigation is the relevance of macrophage polarization to MEC phenotypes. In the simplest terms, macrophages can be polarized to M1 (inflammatory) or M2 (anti-inflammatory) phenotypes, and this polarization changes over time during Cn infection (19). Here we have examined the MEC phenotype using only unpolarized macrophages. While preliminary data suggest that macrophage polarization does modify MECs virulence (TJC and JMD), a role for Pdr802 in these changes remains to be determined.

Our findings demonstrate the utility of MEC induction as an assay with which to dissect Cn pathogenesis and shed new light on how Pdr802 drives virulence despite the lack of *in vitro* phenotypes in the *pdr802*∆ mutant.

## SUPPLEMENTAL MATERIAL

Data S1 spectrum.01398-25-s0001.xlsx Source data for RNA-seq analysis of WT TC and MEC and *ada2*∆ TC and MEC conditions.

Supplementary Data spectrum.01398-25-s0002.docx Detailed methods and Fig. S1.

ASM does not own the copyrights to Supplemental Material that may be linked to, or accessed through, an article. The authors have granted ASM a non-exclusive, world-wide license to publish the Supplemental Material files. Please contact the corresponding author directly for reuse.
