# Supplementary material for: Ada2 acts upstream of Pdr802 in regulating macrophage-enhanced virulence of Cryptococcus neoformans
Source: Microbiol Spectr. 2025 Aug 11;13(9):e01398-25. doi: 10.1128/spectrum.01398-25 (PMC12403825; doi:10.1128/spectrum.01398-25)
Supplement: Supplementary Data — Detailed methods and Fig. S1. [file spectrum.01398-25-s0002.docx]

**SUPPLEMENTAL MATERIAL**

**Detailed Methods**

**Zebrafish care and maintenance.** Adult zebrafish were kept under a light/dark cycle of 14 h and 10 h, respectively. Larval zebrafish were incubated at 28.5°C in E3 buffer [1], switching larvae to E3-MB containing 0.2 nM 1-phenyl-2-thiourea (PTU) (Sigma-Aldrich) at 18-24 hours post fertilization to inhibit pigment formation. All larvae were manually dechorionated between 24 and 30 hpf. Prior to microinjection or imaging, larvae were anesthetized in E3-MB containing 0.2 mg/ml Tricaine (ethyl 3-aminobenzoate; Sigma-Aldrich). For prolonged time lapse imaging, larvae were mounted in 1% low-melting point agarose, 0.2 mg/ml Tricaine, and 0.2nM PTU (final concentrations) on a coverglass-bottom dish. All adult and larval zebrafish procedures were in full compliance with NIH guidelines and approved by the University of Iowa Institutional Animal Care and Use Committee (Protocol #0102075-002).

**Cryptococcal strains and growth conditions.** Library strains H99s (parental), ADA2-1, ADA2-2 and PDR802-1 [2] were kindly provided by Dr. Damian Krysan. Cultures were handled using standard techniques and media as described previously [3, 4].

**Construction of fluorescent Cryptococcus neoformans strains.** WT H99s and *ada2∆* mutant strains with constitutive expression of eGFP (JMD388 and JMD398) were generated from both H99s, ADA2-1 and ADA2-2 library strains [2] using CRISPR, as previously described for the construction of the JMD168 [5].

***Cryptococcus* macrophage transformation.** The mouse macrophage cell line J774A.1 (ATCC TIB-67) was kindly provided by Dr. Melanie Wellington. A confluent T-75 flask of cells exhibiting normal morphology and cell adhesion was split 1:2 in 15 mL DMEM supplemented with 10% FBS, 1% Glutamax, and 1% penicillin streptomycin and allowed to grow overnight. Cryptococcus strains were cultured in 50 mL liquid YPD overnight. The Cryptococcus cells were washed three times in sterile DPBS. 2x10^9^ Cryptococcus cells/mL were opsonized with the 18B7 monoclonal antibody (provided by Dr. Damian Krysan) for 30 minutes prior to co-incubation with macrophages. J774A.1 medium was replaced with 15 mL fresh medium and 5x10^8^ Cryptococcus cells (MOI 20), followed by a 3 hour incubation at 37°C with 5% CO2. Simultaneously, 5x10^8^ Cryptococcus cells were added to 15 mL J774A.1 culture medium in a T-75 culture flask and incubated at 37°C with 5% CO2 as a control. The macrophage/Cryptococcus co-culture was then washed 3 times with warm DPBS to remove non-adherent yeast, then replenished with 15 mL fresh J774A.1 medium and co-cultures and controls were incubated overnight at 37°C with 5% CO2. To lyse the macrophages, 5 mL 10% Triton X-100 in DPBS (final concentration of 2.5%) was added to the co-culture and the control flask then incubated for 20 minutes at room temperature while rocking. Control cultures were processed identically. Cryptococcus cells were then centrifuged and washed twice with sterile DPBS before use.

***Cryptococcus* phagocytosis survival assay.** A confluent T-75 flask of J774.A1 cells exhibiting normal morphology and cell adhesion was split 1:2 in 15 mL DMEM supplemented with 10% FBS, 1% Glutamax, and 1% penicillin streptomycin and allowed to grow overnight. Cryptococcus strains were cultured in 50 mL liquid YPD overnight. The Cryptococcus cells were washed three times in sterile DPBS. 2x10^9^ Cryptococcus cells/mL were opsonized with the 18B7 monoclonal antibody for 30 minutes prior to co-incubation with macrophages. J774A.1 medium was replaced with 15 mL fresh medium and 5x10^8^ Cryptococcus cells (MOI 20), followed by a 3 hour incubation at 37°C with 5% CO2. Initial inoculum was confirmed by dilution plating on YPD medium. The macrophage/Cryptococcus co-culture was then washed 3 times with warm DPBS to remove non-adherent yeast, then replenished with 15 mL fresh J774A.1 medium and co-cultures and controls were incubated overnight at 37°C with 5% CO2. After overnight incubation, co-culture was washed 4 times with warm DPBS and macrophages were lysed using 1% Triton X-100 in DPBS. After an additional wash with DPBS viable yeast were quantified by plating of serial dilutions.

**RNA-Seq and differential expression analysis.** MECs and tissue culture controls derived from indicated strains were washed and diluted to 7.5 x 10^5^ cell/mL. Total RNA was isolated from harvested cells according to manufacturer instructions with an invitrogen PureLink RNA mini-kit (catalog no. 12183018 A, ThermoFisher) with on-column DNAse treatment (catalog no. 12185010, ThermoFisher). Biological triplicates were harvested for each strain and condition. Total RNA (>2 µg per sample) was submitted to Azenta Life Sciences for standard RNA-Seq next-generation sequencing. The RNA samples were quantified using a Qubit 2.0 Fluorometer (ThermoFisher) and RNA integrity was checked using TapeStation (Agilent Technologies). The RNA sequencing libraries were prepared using the NEB Next Ultra II RNA Library Prep Kit from Illumina using the manufacturer’s instructions (New England Biolabs). Briefly, mRNAs were initially enriched with Oligod(T) beads. Enriched mRNAs were fragmented for 15 minutes at 94°C. First-strand and second-strand cDNA were subsequently synthesized. cDNA fragments were end repaired and adenylated at 3’ ends, and universal adapters were ligated to cDNA fragments, followed by index addition and library enrichment by PCR with limited cycles. The sequencing libraries were validated on the Agilent TapeStation (Agilent Technologies) and quantified using a Qubit 2.0 fluorometer (ThermoFisher) as well as by quantitative PCR (KAPA Biosystems). The sequencing libraries were multiplexed and clustered into a flowcell. After clustering, the flowcell was loaded into the Illumina HiSeq instrument according to the manufacturer’s instructions. The samples were sequenced using a 2 x 150 bp Paired End configuration. Image analysis and base calling were conducted by the HiSeq Control Software. Raw sequence data (.bcl files) generated from Illumina HiSeq was converted into fastq files and de-multiplexed using Illumina bcl2fastq 2.20 software. One mismatch was allowed for index sequence identification. Paired-end Illumina sequence read files were evaluated for quality and the absence of adaptor sequence using FastQC (<https://www.bioinformatics.babraham.ac.uc/projects/fastqc/>). Read files were mapped to *C neoformans* reference genome H99 v48 (FungiDB) and gene transcript expression was quantified using HISAT2 and Stringtie [6]. Differential expression fold change, Wald test *p* values and Benjamini-Hochberg adjustment for multiple comparisons were determined using DESeq2. Principle component analysis was performed on regularized log transformed gene counts to confirm the absence of batch effects [7].Gene subset correlation and analysis was performed using FungiDB (VEuPathDB) [8, 9] by paid subscription. GO term analysis was performed using FungiDB and plotted using Revigo [10].

**Zebrafish infection by microinjection.** Cryptococcus cells were macrophage experienced or culture medium experienced as described above. All cultures were diluted in DPBS containing 10% glycerol and 2% PVP-40 (polyvinylpyrrolidine, Sigma Aldrich) [11] to an OD600 of 5.0 in a 1:10 dilution of phenol red. After manual dechorionation of embryos at ~28 hpf, IV inoculations were performed as previously described [12], with the alteration that larvae were positioned on a 3% agarose plate formed with holding grooves as described in [1]. Initial inoculum was documented by direct microscopic observation and only larvae with initial inoculum between ~30 and 70 fluorescent yeast cells were used.

**Pooled zebrafish virulence assay.** 15 larvae per condition were inoculated as above and distributed in a 48 well culture dish in E3 medium. At 2dpi 10 larvae per condition were pooled and euthanized using Tricaine at 250mg/L, pH 7.0. Excess liquid was drawn off and pools of larvae resuspended in 90µl 500µg/mL ampicillin, 500µg/mL kanamycin in DPBS in a microfuge tube. This suspension was then homogenized in a Mini Beadbeater (Biospec Products) at maximum speed. Tubes were left at room temperature for at least 30 minutes to settle and CFU per tube determined by serial dilutions with six technical replicates per dilution.

**Microscopy.** Imaging was performed on a Zeiss Axio Observer.Z1/7 body equipped with a Hamamatsu Flash4.0 V3 sCMOS camera. Fluorescence excitation was generated with a Colibri 7 type RGB-UV fluorescence light source. Filter set was Zeiss set 90 LED. Manual counting of fluorescent cryptococcal cells was performed using a Zeiss 40x/0.75 Plan Neofluar objective. Cryptococcal India ink images (Figures 2E and F) were captured using a Zeiss LD C-Apochromat 60x/1.1 Oil Korr UV VIS IR objective. Cell body diameter and capsule thickness measurements were performed on India Ink preparations using Zeiss Zen software.

**Data Display and Statistical Analysis.** Analysis and graphing was performed using GraphPad PRISM (GraphPad). Image compilation and formatting was performed using Adobe Illustrator (Adobe).

**Supplementary Data 1**. Source data for RNA-seq analysis of WT TC and MEC and *ada2∆* TC and MEC conditions.


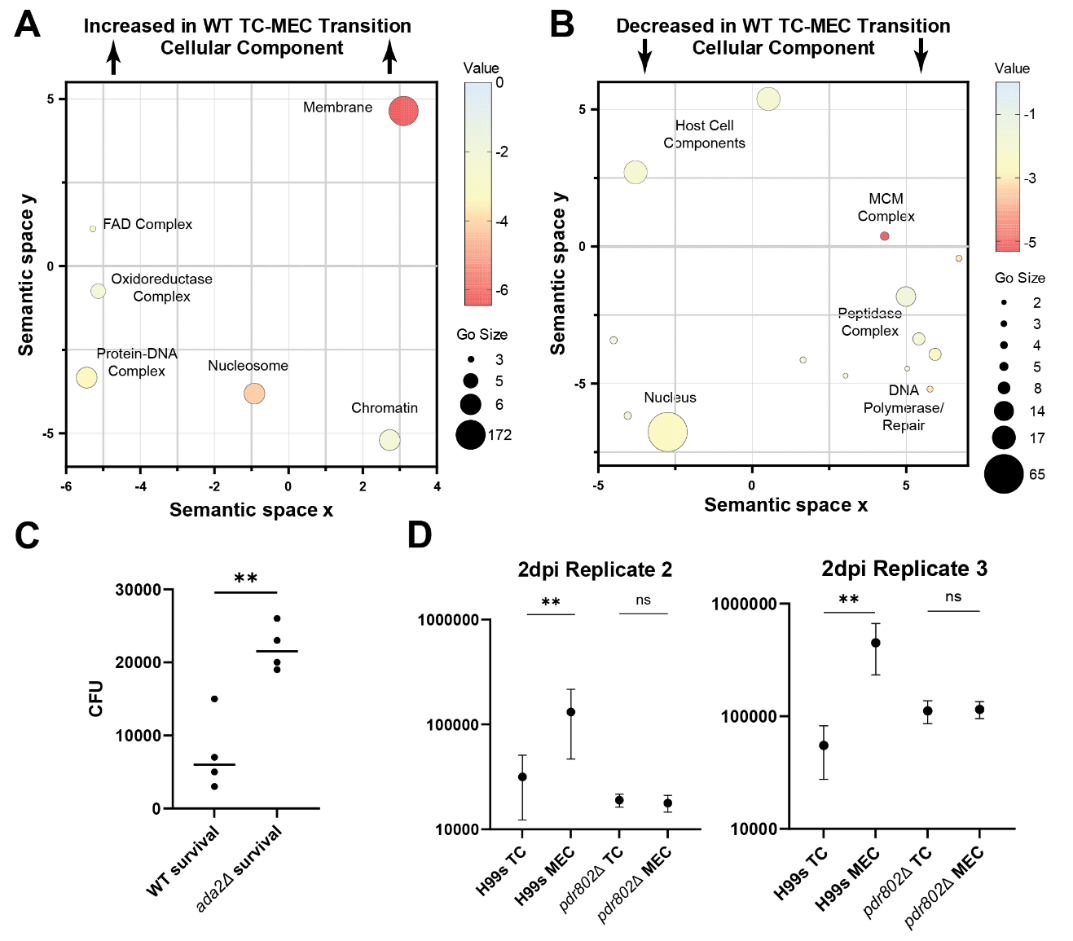


**Supplementary Figure S1.** **A.** and **B.** Cellular Component GO terms for differentially expressed genes (+/-log2 1 and FDR < 0.05, Benjamini-Hochberg) represented in semantic similarity scatterplots for increased and decreased genes, respectively. **C.** Quantification of parental H99s and mutant *ada2∆* strain survival after 48 hours in J774A.1 macrophages. Results of Welch’s t test shown. **D.** Cryptococcal CFU per pool of 10 infected larvae at 2dpi, larvae at 2dpi, second and third replicates of WT and *pdr802∆* mutant from conditions shown. Results of Welch’s t test shown.

**References Cited**

1. Westerfield M. The zebrafish book. A guide for the laboratory use of zebrafish (*Danio rerio*). . Eugene, OR: University of Oregon Press; 2000.

2. Jung KW, Yang DH, Maeng S, Lee KT, So YS, Hong J, et al. Systematic functional profiling of transcription factor networks in Cryptococcus neoformans. Nat Commun. 2015;6:6757. Epub 20150407. doi: 10.1038/ncomms7757. PubMed PMID: 25849373; PubMed Central PMCID: PMCPMC4391232.

3. Davis JM, Huang M, Botts MR, Hull CM, Huttenlocher A. A Zebrafish Model of Cryptococcal Infection Reveals Roles for Macrophages, Endothelial Cells, and Neutrophils in the Establishment and Control of Sustained Fungemia. Infect Immun. 2016;84(10):3047-62. Epub 2016/08/03. doi: 10.1128/IAI.00506-16. PubMed PMID: 27481252; PubMed Central PMCID: PMCPMC5038067.

4. Sherman F. Getting started with yeast. Methods Enzymol. 2002;350:3-41. doi: 10.1016/s0076-6879(02)50954-x. PubMed PMID: 12073320.

5. Nielson JA, Davis JM. Roles for Microglia in Cryptococcal Brain Dissemination in the Zebrafish Larva. Microbiol Spectr. 2023;11(2):e0431522. Epub 2023/02/01. doi: 10.1128/spectrum.04315-22. PubMed PMID: 36719205; PubMed Central PMCID: PMCPMC10100726.

6. Pertea M, Kim D, Pertea GM, Leek JT, Salzberg SL. Transcript-level expression analysis of RNA-seq experiments with HISAT, StringTie and Ballgown. Nat Protoc. 2016;11(9):1650-67. Epub 20160811. doi: 10.1038/nprot.2016.095. PubMed PMID: 27560171; PubMed Central PMCID: PMCPMC5032908.

7. Love MI, Anders S, Kim V, Huber W. RNA-Seq workflow: gene-level exploratory analysis and differential expression. F1000Res. 2015;4:1070. Epub 20151014. doi: 10.12688/f1000research.7035.1. PubMed PMID: 26674615; PubMed Central PMCID: PMCPMC4670015.

8. Amos B, Aurrecoechea C, Barba M, Barreto A, Basenko EY, Bazant W, et al. VEuPathDB: the eukaryotic pathogen, vector and host bioinformatics resource center. Nucleic Acids Res. 2022;50(D1):D898-D911. doi: 10.1093/nar/gkab929. PubMed PMID: 34718728; PubMed Central PMCID: PMCPMC8728164.

9. Basenko EY, Pulman JA, Shanmugasundram A, Harb OS, Crouch K, Starns D, et al. FungiDB: An Integrated Bioinformatic Resource for Fungi and Oomycetes. J Fungi (Basel). 2018;4(1). Epub 20180320. doi: 10.3390/jof4010039. PubMed PMID: 30152809; PubMed Central PMCID: PMCPMC5872342.

10. Supek F, Bosnjak M, Skunca N, Smuc T. REVIGO summarizes and visualizes long lists of gene ontology terms. PLoS One. 2011;6(7):e21800. Epub 20110718. doi: 10.1371/journal.pone.0021800. PubMed PMID: 21789182; PubMed Central PMCID: PMCPMC3138752.

11. Vincent WJ, Freisinger CM, Lam PY, Huttenlocher A, Sauer JD. Macrophages mediate flagellin induced inflammasome activation and host defense in zebrafish. Cell Microbiol. 2016;18(4):591-604. Epub 20151104. doi: 10.1111/cmi.12536. PubMed PMID: 26468080; PubMed Central PMCID: PMCPMC5027955.

12. Cosma CL, Swaim LE, Volkman H, Ramakrishnan L, Davis JM. Zebrafish and frog models of Mycobacterium marinum infection. Curr Protoc Microbiol. 2006;Chapter 10:Unit 10B 2. doi: 10.1002/0471729256.mc10b02s3. PubMed PMID: 18770575.
